# Virulence Profiles and Antibiotic Susceptibility of *Escherichia coli* Strains from Pet Reptiles

**DOI:** 10.3390/pathogens11020127

**Published:** 2022-01-21

**Authors:** Marta Dec, Dagmara Stepien-Pysniak, Klaudiusz Szczepaniak, Barbara Turchi, Renata Urban-Chmiel

**Affiliations:** 1 Department of Veterinary Prevention and Avian Diseases, Faculty of Veterinary Medicine, University of Life Sciences in Lublin, Akademicka 12, 20-033 Lublin, Poland; dagmara.stepien@up.lublin.pl (D.S.-P.); renata.urban@up.lublin.pl (R.U.-C.); 2 Department of Parasitology and Fish Diseases, Faculty of Veterinary Medicine, University of Life Sciences in Lublin, Akademicka 12, 20-033 Lublin, Poland; klaudiusz.szczepaniak@up.lublin.pl; 3 Department of Veterinary Science, University of Pisa, Viale delle Piagge 2, 56124 Pisa, Italy; barbara.turchi@unipi.it

**Keywords:** reptiles, *E. coli*, virulence genes, antibiotic susceptibility, phylogenetic group, rep-PCR

## Abstract

Exotic reptiles are increasingly being bred as pets in many countries around the world, including Poland. However, the close contact between reptiles and their owners provides favourable conditions for the transmission of zoonotic pathogens. In this work, we examined *E. coli* isolates from 67 captive reptiles regarding their virulence, antibiotic susceptibility, phylogenetic affiliation, and genetic diversity. The incidence of *E. coli* was highest in snakes (51.6%, 16 isolates/31 samples), and slightly lower in turtles (44.4%, 8/18) and lizards (44.4%, 8/18). Genes encoding virulence factors were confirmed in 50% of isolates and the most common were the *traT* (37.5%, *n* = 12), *fyuA* (21.87%, *n* = 7), and *irp-2* (15.62%, *n* = 5). The majority (71.87%, *n* = 23) of *E. coli* isolates were susceptible to all of the antimicrobial substances used in the study. Streptomycin resistance (21.87%, *n* = 7) was the most frequent, while resistance to other antimicrobial substances was sporadic. One strain (3.12%) was classified as multidrug-resistant. The presence of resistance genes (*aadA, tetA, tetB, tetM,* and *blaTEM*) was confirmed in 12.5% (*n* = 4) of the isolates. The majority (65.6%, *n* = 21) of *E. coli* isolates represented the B1 phylogenetic group. (GTG)_5_-PCR fingerprinting showed considerable genetic variation in the pool of tested isolates. The frequency of *E. coli* in reptiles is much lower than in mammals or birds. Due to the presence of virulence genes, characteristic of both intestinal pathogenic *E. coli* (IPEC) and extraintestinal pathogenic *E. coli* (ExPEC), reptilian strains of *E. coli* have pathogenic potential, and therefore people in contact with these animals should follow good hygiene practices.

## 1. Introduction

In the last few years, exotic reptiles have risen in popularity as pets, with a population of over 9 million in European households. The estimated number of captive reptiles in Poland is 215,000 [1] (pp. 44, 50). The corn snake, ball python, steppe tortoise, Greek tortoise, and lizards such as the bearded agama, chameleon and geckos are very popular [2]. Pet reptiles are sometimes kept in terrariums, but often they are free to move about in homes and treated as companion animals. In addition, reptile exhibitions organized in public spaces provide opportunities not only to observe these animals, but also to touch or hold them. However, close contact between reptiles and humans poses a public health risk, as these animals may harbour and excrete potentially pathogenic microorganisms. Reptiles are well recognized as asymptomatic carriers of *Salmonella*, including serotypes which can cause infections in humans, known as reptile-associated salmonellosis (RAS). At the same time, data on the characteristics of reptilian *E. coli* strains (RepEC), which, similarly to *Salmonella*, belong to the *Enterobacteriaceae* family, are very limited [3,4].

Within the *E. coli* species, apart from commensals commonly colonizing the intestines of mammals and birds, there are also intestinal pathogenic *E. coli* (IPEC) and extraintestinal pathogenic *E. coli* (ExPEC) strains. The formers are diarrhoeagenic pathogens, and the latter colonize other parts of the host’s body but can also exist as commensals. Each of these two groups contains a number of pathotypes that differ in their range of virulence factors and the type of disease they cause. However, *E. coli* has high genome plasticity, and hybrid strains carrying a combination of both IPEC and ExPEC virulence-associated genes are known as well [5]. ExpPEC strains, i.e., avian pathogenic *E. coli* (APEC), uropathogenic *E. coli* (UPEC), and neonatal meningitis *E. coli* (NMEC), are responsible for many diseases of humans and animals, such as urinary tract infections (UTIs), pneumonia, meningitis, diverse intra-abdominal infections, soft tissue infections, osteomyelitis, and sepsis [6]. Due to the high genetic similarity of virulent ExpPEC strains from animals and humans, it has been suggested that livestock or pets may be a reservoir for the transmission of *E. coli* infections [7,8]. Among IPECs, the enterohaemorrhagic *E. coli* (EHEC) strains that produce Shiga toxin are considered the most dangerous for humans. The symptoms of EHEC infection are bloody diarrhoea and severe colitis, and some patients develop haemolytic uraemic syndrome (HUS), which can be fatal [9]. The main reservoirs of Shiga-toxin-producing strains of *E. coli* are ruminants (cattle, sheep, and goats), but these strains have also been detected in reptiles. Moreover, lizards can be carriers of other IPECs as well, i.e., enteroaggregative *E. coli* (EAEC), enterotoxigenic *E. coli* (ETEC), and enteropathogenic *E. coli* (EPEC) [9,10].

Bearing in mind the above, as well as the growing interest in reptile breeding and the lack of information on the occurrence and characteristics of RepEC, in this study we aimed to determine the prevalence of *E. coli* strains in captive reptiles and their virulence and antibiotic sensitivity profiles, as well as their phylogenetic affiliation and genetic variation. Monitoring the presence of microorganisms that are dangerous to humans, such as pathogenic and drug-resistant *E. coli*, and identifying reservoirs of such strains is important for assessing the risk associated with their spread and possible infection. 

## 2. Results

### 2.1. Identification of E. coli 

The identification of all putative *E. coli* isolates grown on MacConkey medium was confirmed by PCR by using two marker genes, i.e., *uidA* and *uspA*. Glucuronidase production was also confirmed in all isolates by a phenotypic test using TBX agar. The presence of *E. coli* was recorded in 32 of 67 faecal samples (47.8%). A total of 32 strains of *E. coli* were isolated, each from a different stool sample. The incidence of *E. coli* was highest for snake samples (51.6%, 16 isolates/31 samples) and slightly lower for turtles (44.4%, 8/18) and lizards (44.4%, 8/18) (Table 1); however, these differences were not statistically significant (P = 0.881, chi2 = 0.253, contingency coefficients = 0.06). There were also no significant differences between the frequency of *E. coli* in carnivorous (48.5%, 16 isolates/33 samples), herbivorous (45.4%, 10/22) and omnivorous (54.5%, 6/11) reptiles (P = 0.886, chi2 = 0.243, contingency coefficients = 0.06).

### 2.2. Detection of Virulence-Associated Genes 

Genes encoding virulence factors were detected in 50% (*n* = 16) *E. coli* strains. The most commonly found were *traT* (found in 37.5% of isolates, *n* = 12), encoding complement resistant protein, *fyuA* (21.87%, *n* = 7), coding for the ferric yersiniabactin uptake receptor, and *irp-2* (15.62%, *n* = 5), responsible for biosynthesis of the siderophore yersiniabactin. Other virulence genes were detected less frequently, i.e., *vat* (vacuolating autotransporter toxin, 9.37%, *n* = 3), *pic* (serine protease *pic* autotransporter, which shows proteolytic activity on complement system proteins, 6.25%, *n* = 2), *iucD* (aerobactin siderophore synthesis, 3%, *n* = 1) and *astA* (enteroaggregative *E. coli* heat-stable toxin EAST1, 3.12%, *n* = 1) (Table 2). 

In seven isolates, the coexistence of between two and four virulence genes was recorded and, interestingly, as many as six of these strains were obtained from snakes (Table 2). Moreover, 1 strain (16a, from a corn snake) with a *pic-astA* profile can be classified as IPEC, 1 strain (41, from milk snake) with a *pic-irp-2-traT-fyuA* profile is a hybrid strain (the *pic* gene is characteristic of IPEC, and *irp-2, traT* and *fyuA* for ExPEC), while the remaining strains contain virulence genes characteristic of ExPEC. No *E. coli* strain contained any of the remaining virulence genes characteristic of either IPEC (*stx1, stx2, hlyA, eaeA, saa, escV, ent, bfpB, elt, estIa, estIb, invE, ipaH, and aggR*) or ExPEC (*pap-C, ompT, cva/cvi, iss, iutA, and kpsII*).

### 2.3. Antibiotic Susceptibility Testing 

Most of the *E. coli* isolates (71.87%, 23/32) were found to be susceptible to all of the tested antimicrobial agents. Among the remaining isolates, the phenotype of resistance to streptomycin (21.87%, 7/32) was the most frequent, while resistance to other substances, i.e., gentamicin (6.25%, 2/32), amikacin (6.25%, 2/32), tetracycline (6.25%, 2/32), ampicillin (3.12% 1/32), amoxicillin/clavulanic acid (3.12%, 1/32), and trimethoprim (3.12%. 1/32), was observed much less frequently. A total of 3 strains (9.37%) showed resistance to more than 1 drug, but only 1 strain (3.125%), *E. coli* 37a from corn snake, was classified as multidrug-resistant (MDR, defined as resistance to at least 3 or more antibiotics belonging to different antimicrobial categories). It is also interesting that intermediate streptomycin susceptibility was recorded for the majority of the strains tested (59.37%, 19/32). The 2 strains that showed resistance to AMC or AMP and 4 strains with intermediate susceptibility to AMP were susceptible to cephalosporins (2G, 3G and 4G) and carbapenems (Table 2).

### 2.4. Detection of Resistance Genes

The occurrence of the resistance genes was recorded for only 4 (12.5%) *E. coli* strains. Moreover, 1 strain (3.12%) was detected with the *aadA* gene encoding streptomycin/spectinomycin adenylyltransferase, 2 strains (6.25%) were confirmed with the *tetA*, *tetB* and *tetM* genes conferring resistance to tetracyclines, and 1 isolate (3.12%) carried the TEM-type beta-lactamase (*bla*TEM). For each of these strains, a correlation was found between the phenotype and the presence of resistance genes (Table 2).

### 2.5. Determination of E. coli Phylogenetic Groups

**Figure 1:**
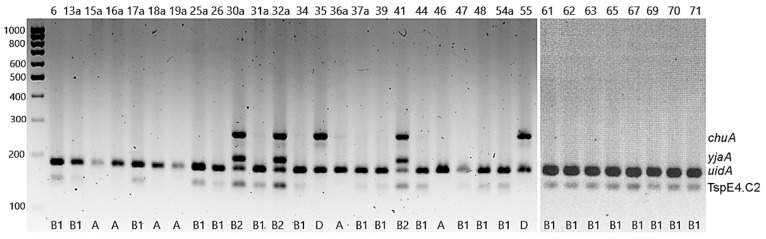
Multiplex PCR patterns for *E. coli* phylogenetic groups.

Phylogenetic groups of *E. coli* were determined based on the electrophoretic profiles of multiplex PCR amplicons (*yja*, TspE4.C2, *chuA*, *svg* and *uidA*) (Figure 1). Over 65% of the isolates (65.6%, *n* = 21) belonged to group B1, 18.7% (*n* = 6) were assigned to group A, 9.4% (*n* = 3) to B2, and 6.2% (*n* = 2) to group D. None of the isolates belonged to group B2_1_ (no isolate contained the *svg* gene) (Figure 1). It is worth noting that all the strains representing group B2 were collected from snakes (Table 2).

### 2.6. Rep-PCR Fingerprinting 

**Figure 2:**
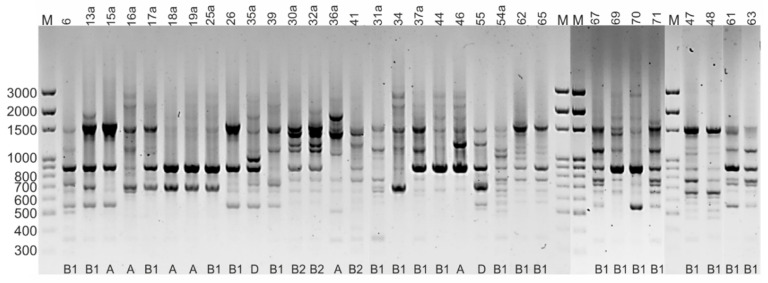
Agarose gel patterns of products amplified by (GTG)_5_-PCR.

**Figure 3:**
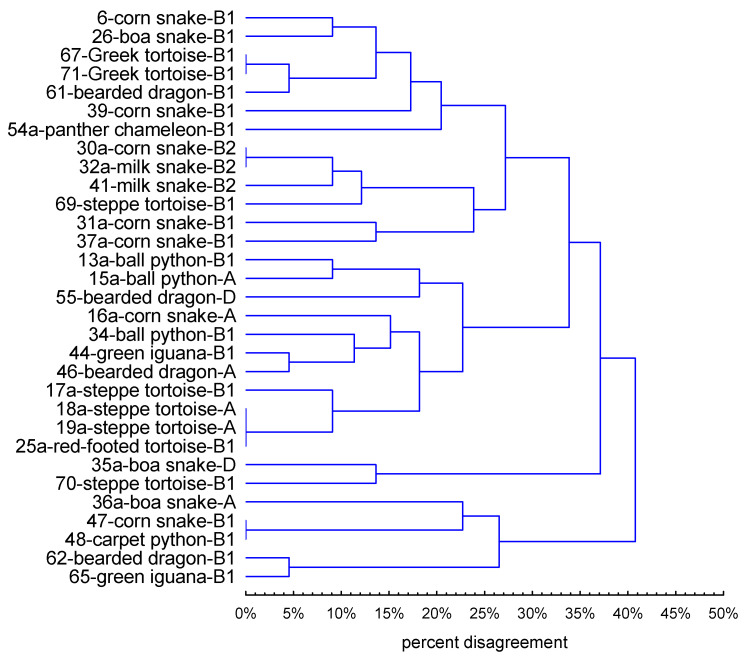
Dendrogram generated by UPGMA clustering from (GTG)_5_-PCR fingerprinting of *E. coli* strains.

DNA amplification with the (GTG)_5_ primer revealed significant genotypic diversity among the *E. coli* isolates. The electrophoretic profiles were reproducible in the 2 separate PCR reactions, and the fingerprinting generated distinct amplification bands ranging in size from 350 bp to 2700 bp. The profiles obtained contained between 2 and 12 PCR products and allowed the 32 strains to be grouped into 27 rep-types (Figure 2). This indicates considerable genetic diversity of the *E. coli* isolates, with a maximum percentage of dissimilarity of 41%. There was no correlation between the electrophoretic profile and the phylogenetic group or the host species, although several strains representing the same phylogenetic group had identical fingerprints (Figure 3).

## 3. Discussion

The scientific literature abounds with information on the drug susceptibility and virulence of *E. coli* isolated from humans, mammals, and birds, but reports on the occurrence and characteristics of *E. coli* in reptiles are scarce. The prevalence of the *E. coli* strains reported in these studies (47.8%) is in line with that reported by Gopee et al. [11] (52%) for reptiles from the Emperor Valley Zoo, Trinidad, Spain. The authors recorded the highest incidence of *E. coli* in Crocodylidae (83.3%), lower incidences in turtles (68.08%) and snakes (37.5–51.1%, depending on the family), and the lowest for lizards (33.3%). In contrast with our observations, a higher frequency of *E. coli* was recorded in herbivorous reptiles (66.7%) than in carnivorous reptiles (46.4%).

A significantly higher frequency of *E. coli* in captive and free-living reptiles was recorded by Ramos et al. [3] in Brazil (68%) and, as in our study, they reported a higher incidence of *E. coli* in snakes (84.4%) than in lizards (46.6%) and turtles (40–50%). Similar results were obtained by Sylvester et al. [12] and Bautista-Trujillo et al. [10], who showed the presence of *E. coli* in approximately 40% of green iguanas. Several other authors have also described the presence of *E. coli* in reptiles; however, they did not take into account the frequency of this bacterium in individual groups of reptiles. Książczyk et al. [4] isolated a total of 35 *E. coli* strains from 103 stool samples from reptiles at the zoo in Wrocław, Poland. These data indicate 34% prevalence, assuming each strain was isolated from a different sample. Much higher prevalence of *E. coli* in reptiles is suggested by the findings of Unger et al. [13], who isolated a total of 142 *E. coli* isolates from 150 faecal samples derived from >60 exotic reptile species crossing the veterinary border at Frankfurt airport.

Warm-blooded animals (mammals and birds) are already known to be reservoirs of both IPEC and ExPEC *E. coli* strains [14,15,16], but knowledge of the occurrence of pathogenic *E. coli* in cold-blooded animals is scant. In terms of the presence of virulence genes, our research is largely in line with the findings of Ramos et al. [3], who detected only 2 of the many virulence genes characteristic for ETEC, EHEC and NTEC, i.e., the *cnf1* gene encoding cytotoxic necrotizing factor 1 (found in 9.2% of RepEC isolates) and the *astA* gene encoding EAST1 toxin (2.6%). The frequency described in this study (3.12%) of the *astA* gene, coding for heat-stable enterotoxin EAST1 characteristic of EAEC, is also consistent with results reported by Książczyk et al. [4]. In the case of virulence genes characteristic for IPEC, it should be emphasized that our research is the first report on the presence of the *pic* gene in RepEC, characteristic for the EHEC pathotype. Completely different results were presented by Bautista-Trujillo et al. [10], who classified 62 of 100 *E. coli* isolates from captive green iguanas in Mexico as diarrhoeagenic *E. coli* (DEC) strains. Among them, STEC strains (40.3%) carrying the *stx1* (38.7%) or *stx2* (1.6%) gene were the most prevalent pathotype, followed by EAEC (27.4%, *aap*-positive strains), ETEC (27.4% each, *lt*- or *st*-positive) and EPEC (4.9%, *bfpA*- or *eaeA*-positive). The frequency of *tratT* (37.5%, coding for complement resistance protein) and *fyuA* (21.87%, coding for the ferric yersiniabactin uptake receptor) genes reported in these studies was about twice as high as in the case of RepEC strains isolated in Poland (*traT*-16.7%, *fyuA*-8.3%) by another research team [4]. The *fyuA* gene is common in both UPEC and APEC strains, while the *traT* gene is characteristic of the UPEC pathotype [4]. The occasional presence of the *irp-2* gene in RepEC (8.3%) was also demonstrated by Książczyk et al. [4]. However, in contrast to our results, they did not detect the *iucD* or *vat* gene in any strain, and in several strains, they confirmed the presence of other virulence genes characteristic of ExPEC, i.e., *papC*, *iss* and *tsh*.

The results of this study on drug resistance in *E. coli* most closely match the findings of Sylvester et al. [12], who showed that among *E. coli* strains from wild and pet green iguanas (from Grenada), resistance to streptomycin (12%) and amoxicillin-clavulanic acid (12%) was the most common, and only 7% of strains were multi-drug resistant. A much higher prevalence of resistant *E. coli* strains was recorded in reptiles housed at zoo in Trinidad [11], and resistance to ampicillin (66.7%), tetracycline (57.6%) and gentamicin (12.1%) was the most frequent. Interestingly, the prevalence of streptomycin resistance (36.4%) demonstrated by these studies was somewhat similar to our data (21.9%). An agreement was also noted in the lack of resistance to fluoroquinolones. A high percentage of resistant *E. coli* strains was also demonstrated by Bautista-Trujillo et al. [10], who classified as many as 82.3% of DEC isolates (from captive green iguanas in Mexico) as MDR, and most frequently noted resistance to amikacin (~40%) and ampicillin (~30%). 

The higher incidence of drug-resistant strains in captive reptiles from Trinidad and Mexico may be due to different veterinary practices and the different prevalence of drug-resistant *E. coli* in the reptile diet and environment. Details on the type and frequency of antibiotic use in reptiles in these countries are not known, but Mexico was among the top ten countries in the world with the highest use of antibiotics in veterinary medicine in 2017 [16].

The higher frequency of resistance to streptomycin (~22%) compared to resistance to other antimicrobial substances (0–6.25%) reported in these studies may be due to the widespread use of this antibiotic in veterinary medicine as well as in horticulture as a plant protection product. The resulting streptomycin-resistant strains may spread in the environment and eventually be acquired by the reptiles via their feed (plants, rodents, meat). Moreover, the results obtained in this study and some literature data suggest that the reptilian *Enterobacteriaceae* may have a reduced susceptibility to streptomycin. Streptomycin resistance was the most frequently reported antibiotic resistance phenotype in reptile-derived *Salmonella* strains in Poland (25%) [17] and Indonesia (75%) [18]. Similarly, Bertolini et al. [19] showed a high percentage (79%) of *Salmonella* strains intermediately susceptible to streptomycin in reptiles.

According to our best knowledge, this work is the first full report of the occurrence of resistance genes in *E. coli* from reptiles. The *aadA* (aminoglycoside (3″) (9) adenylyltransferase), *tetA* (tetracycline efflux protein TetA), *tetB* (tetracycline efflux protein TetB) and *bla*TEM genes detected in several RepEC strains are commonly found in drug-resistant *Enterobacteriaceae* isolated from humans and farm animals [20,21]. The *tetM* gene coding for tetracycline ribosomal protection protein has thus far been found sporadically in *E. coli*, mainly in pig and chicken strains [21]. Its presence confirmed in this work in one RepEC isolate may be the result of genetic transfer from *Enterococcus* or *Lactobacillaceae*, common carriers of *tetM* [22,23]. The TEM family of β-lactamases covers a large group of enzymes and determining which TEM lactamase has been detected in these studies requires more detailed sequencing-based analyzes. In *E. coli* strains, the most common is the prototype narrow-spectrum *bla*TEM-1 lactamase, from which the broad-spectrum lactamases (ESBL) have evolved by mutation [24,25]. The *bla*TEM-1 gene was also found in two strains of *E. coli* from reptiles transported across the veterinary border in Frankfurt, where it coexisted with many other resistance genes [13].

Phylogenetic group B1, which dominated (65.6%) among the RepEC isolates, is characteristic of non-pathogenic *E. coli* strains, while groups A and B2, recorded in this study for a total of 9 isolates, is characteristic of ExPEC, i.e., UPEC and APEC [4,26]. It is therefore worth noting that 3 of 4 RepEC strains, containing 4 virulence genes simultaneously, belonged to the B2 phylogenetic group. The results presented in this study are consistent with the reports of Gordon and Cowling [27], who assigned nearly 70% of *E. coli* isolates from snakes and lizards to phylogenetic group B1, while strains of groups A, B2 and D accounted for 15.1%, 6.1% and 9.1%, respectively. A higher percentage (25%) of strains representing the B2 phylogenetic group was recorded in turtles [28]. A higher frequency of strains of the B1 group in RepEC was reported by Ramos et al. [3] (88.4%) and Książczyk et al. [4] (95.8%).

## 4. Materials and Methods

### 4.1. Collection of Faecal Samples 

The research material consisted of faecal samples collected between 2017 and 2020 from 67 reptiles, i.e., snakes (*n* = 31), lizards (*n* = 18) and turtles (*n* = 18) living in captivity or as pets. The animals did not show any symptoms of disease. They were obtained from private owners (*n* = 53) and from pet shops (*n* = 14) in the Lubelskie Province, Poland.

### 4.2. E. coli Isolation 

Stool specimens collected with a swab were suspended in peptone water and incubated for approximately 18 hours, inoculated onto MacConkey agar (Oxoid Ltd., Altrincham, UK), and incubated at 37 °C for 24 h under aerobic conditions. Single pink colonies were inoculated on TSB (trypticase soy broth) (Oxoid Ltd., Altrincham, UK), and pure cultures supplemented with 20% glycerol were stored at −80 °C for further analysis. To determine the ability of the strains to produce glucuronidase, they were additionally plated on TXB agar and incubated ~24 h at 44 °C.

### 4.3. Identification of E. coli

Presumptive *E. coli* isolates were identified based on detection of the *uidA* glucuronidase gene and the *E. coli*-specific flanking region of the *uspA* gene (coding for universal stress protein). PCR was performed using DreamTaq polymerase (Thermo Fisher Scientific, Vilnius, Lithuania) and the following two pairs of primers: uidAF-TATGGAATTTCGCCGATTTT and uidAR-TGTTTGCCTCCCTGCTGCGG (*uidA* amplicon size 166 bp) and uspAF-CCGATACGCTGCCAATCAGT and uspAR-ACGCAGACCGTAGGCCAGAT (*uspA* amlicon 884 bp) [25]. The PCR reaction were performed using Dream Taq polymerase and the following thermal-cycling program: initial denaturation at 94 °C for 5 min; 30 cycles of 94 °C for 45 s, 57 °C for 45 s and 72 °C for 1 min; final extension step at 72 °C for 8 min. PCR products (8 µL, ~600 ng) were separated by electrophoresis (100 V, 1 h) on 1.8% agarose gels and visualized by SimplySafe (Eurx, Gdańsk, PL) staining. PCR product sizes were determined by comparison with a Nova 100 bp DNA ladder (Novazym, Poznań, Poland) using Image Lab 6.1. Software (Bio-Rad, Hercules, CA, USA). 

### 4.4. DNA Extraction 

Whole-genome DNA was extracted from all of the tested *E. coli* isolates (*n* = 32) using the Gene MATRIX Bacterial & Yeast Genomic DNA Purification Kit (Eurx, Gdańsk, Popland) following the manufacturer’s instructions and stored at −20 °C. DNA concentration was determined by measuring the absorbance of 2 µL of the sample at 260/280  nm using the NanoDrop Lite spectrophotometer (Thermo Fisher Scientific, Waltham, MA, USA), and the quality of DNA was analyzed by agarose (1.5% *w*/*v*) gel electrophoresis. No signs of DNA degradation (smearing) were observed in any of the samples, and its concentration was ~20 ng/µL.

### 4.5. Detection of Virulence Genes

Uniplex or multiplex PCR (Table 3), using gene-specific primers (Table S1), was used to detect the presence of 29 genes associated with virulence in both intestinal and extraintestinal pathogenic *E. coli* strains. Two *E. coli* strains from wild mammals previously confirmed to have some virulence genes [16], i.e., astA-stx1-stx2-hlyA (*E. coli* 22a) and astA-escV-eaeA (*E. coli* 19), were used as positive controls. All PCR reactions were performed in an Eppendorf Mastercycler using Dream Taq polymerase (Thermo Scientific). PCR products (8 µL, ~600 ng) were separated by electrophoresis (100 V, 1 h) on 1.8% agarose gels and visualized by SimplySafe (Eurx, Gdańsk, Poland) staining. PCR product sizes were determined by comparison with a Nova 100 bp DNA ladder (Novazym, Poznań, Poland) using Image Lab 6.1. Software (Bio-Rad, Herkules, CA, USA).

### 4.6. Antimicrobial Susceptibility Testing 

Antibiotic susceptibility testing was performed by the agar disk diffusion method according to Clinical and Laboratory Standards Institute (CLSI) recommendations [31]. Isolates were revitalized on tryptone soy agar (TSA) plates (Oxoid, Altrincham, UK). Bacterial suspensions with a turbidity equivalent to McFarland Standard 0.5 were swabbed on Müeller Hinton agar plates (Oxoid, Altrincham, UK) with a sterile cotton swab. Antibiotic disks (Oxoid, Altrincham, UK) containing ampicillin (AMP, 10 µg), amoxicillin/clavulanic acid (AMC, 20/10 µg), cefotaxime (CTX, 30 µg), chloramphenicol (C, 30 µg), tetracycline (TE, 30 µg), trimethoprim (W, 5 µg), ciprofloxacin (CIP, 5 µg), streptomycin (S, 10 µg), gentamicin (CN, 10 µg), amikacin (AK, 30 µg) and nitrofurantoin (F, 100 µg) were placed on the plates. Strains that showed resistance or intermediate susceptibility to AMP or AMC were additionally tested against cefoxitin (FOX, 30 µg), cefepime (FEP 30 µg), ceftazidime (CAZ 30 µg), ceftazidime/clavulanic acid (CZC, 30/10 µg), imipenem (IMP, 10 µg) and meropenem (MEM, 10 µg). Inhibition zone diameters, including the diameter of the 6-mm disks, were measured after incubation at 35 °C for 20 h. Isolates were classified as resistant I, intermediate (I), and susceptible (S) according to breakpoints provided by CLSI [31]; only in the case of nitrofurantoin was the interpretation of the results based on EUCAST guidelines [32]. *E. coli* ATCC 25922 reference strain was used as a quality control. 

### 4.7. Detection of Resistance Genes 

All of hte *E. coli* isolates that showed phenotypic resistance or intermediate susceptibility to specific antimicrobial agents were tested for the presence of relevant resistance genes using a uniplex or multiplex PCR assay. Specifically, genes conferring resistance to beta-lactams (*blaTEM, blaOXY, blaCTX and blaSHV*), tetracyclines (*tetA, tetB, tetC, tetK, tetL, tetM* and *tetO*), aminoglycosides (*aadA, strA/strB, aac(3)-II, aac(3)-IV, aphA1 and aphA2*), trimethoprim and sulfonamides (*sul1, sul2, sul3, dfrA1, dfrA5*, and *drfA7-A17*) were detected using the primers and annealing temperatures shown in Table S2. Analysis of the PCR products was performed as above.

### 4.8. Determination of E. coli Phylogenetic Groups

To determine the phylogenetic groups of the *E. coli* isolates, five sets of primers for the five sets of primers for the genes *yja*, TspE4.C2, *chuA*, *svg* and *uidA* (Table S3) were used in a multiplex PCR, as previously described [33]. PCR products were separated by electrophoresis in 2.5% (wt/vol) high resolution agarose (Blirt, Gdańsk, Poland). The amplicons sizes were determined by comparison with a Nova 100 bp DNA ladder (Novazym, Poznań, Poland) using Image Lab 6.1. Software (Bio-Rad, Herkules, CA, USA). The phylogenetic groups were determined based on the PCR gel pattern [33].

### 4.9. Rep-PCR Fingerprinting 

The genetic diversity of *E. coli* isolates was assessed by repetitive extragenic palindromic (REP)-PCR using the (GTG)_5_ primer -5′-GTGGTGGTGGTGGTG-3′ [34]. The PCR reactions were carried out in 18 µL of a reaction mixture containing 7.5 µL of 2x PCR Mix Plus (A&A Biotechnology, Gdynia, Poland), 0.75 µL of (GTG)_5_ primer, 0.75 µL (~20 ng/µL) of DNA template, and 9 µL of H_2_O. Amplification was performed in the following sequence: initial denaturation 5 min, 95 °C; 40 cycles at 95 °C for 45 s, 42 °C for 35 s and 72 °C for 2 min; and final extension at 72 °C for 8 min. PCR products were separated by electrophoresis in 1.5% agarose. The gels were run at 80 V in 1X TBE buffer, stained with SimplySafe (Eurx, Gdańsk, Poland), and visualized under an UV source using the GelDoc Go imaging system (BioRad, Herkules, CA, USA). Profiles were analysed with Image Lab Software (BioRad, Herkules, CA, USA) by comparison with the Nova 100 bp DNA ladder (Novazym, Poznań, Poland).

The presence of a given band was coded as 1 and the absence of a given band was coded as 0 in a data matrix (Excel, Microsoft Office), and the unweighted pair group method with arithmetic averages (UPGMA) was used to generate a dendrogram of dissimilarity in Statistica software (Ver. 13.1, Tulsa, OK, USA).

### 4.10. Statistical Analysis 

Pearson’s chi-squared test was used to determine the relationship between diet (carnivores, omnivores, herbivores) and the frequency of *E. coli*, as well as between the type of reptile (snakes, lizards, turtles) and the frequency of *E. coli* isolation. The level of significance was set as *p* < 0.05. The statistical analysis was performed using Microsoft Excel 2019.

## 5. Conclusions

The results presented in this study contribute to knowledge of the occurrence and phenotypic and genotypic features of RepEC strains. Although the incidence of drug resistance in *E. coli* strains from reptiles in Poland is low, the possibility of transmission of resistance genes to other members of *Enterobacteriaceae*, including *Salmonella*, which commonly inhabits reptile intestines, should be taken into account. In addition, RepEC strains contain virulence genes characteristic of ExPEC, and less often of IPEC. IPEC strains are diarrhoeagenic pathogens, while ExPEC strains may exist as commensals, but under favourable conditions can cause infections. Due to the pathogenic potential of RepEC strains, they may pose a health risk to humans, and reptile keepers should use good hygiene practices when handling these animals.

## Figures and Tables

**Table 1:** Frequency of isolation of *Escherichia coli* from captive reptiles in Poland.

Group of Reptiles	Number of Samples	Species (Number of Samples)	Diet Group	Number of *E. coli* Isolates (%)
Snakes	31	*Pantheropsis guttatus* (14) *Python regius* (3) *Boa constrictor* (4) *Lampropeltis triangulum* (4) *Morelia pilota* (1) *Orthrophis teaniurus* (5)	Carnivore Carnivore Carnivore Carnivore Carnivore Carnivore	7 (50) 3 (100) 3 (75) 2 (50) 1 (100) 0
Lizards	18	*Pogona vitticeps* (6) *Iguana iguana* (4) *Eublepharis macularius* (3) *Furcifer pardali* (5)	Omnivore Herbivore Carnivore Omnivore	5 (83) 2 (50) 0 1 (20)
Turtles	18	*Testudo horsfieldii* (8) *Testudo hermanni* (9) *Chelonoidis carbonaria* (1)	Herbivore Herbivore Herbivore	5 (62.5) 2 (22) 1 (100)
Total:	67			32 (47.8)

**Table 2:** Phenotypic and genotypic drug resistance profiles, virulence profiles and phylogenetic groups of *E. coli* strains isolated from reptiles.

Isolate	Species	Phylogenetic Group	Antibiotic Phenotype Pattern (Including Resistant and Intermediate Strains) ^a^	Resistance Genes	Virulence Genes
6	Corn snake (*Pantheropsis guttatus*)	B1	AMP-AK-**S**-CIP	-	*traT*
13a	Ball python (*Python regius*)	B1	AMP	-	*irp-2*, *iucD*, *traT*, *fyuA*
15a	Ball python (*Python regius*)	A	-	-	-
16a	Corn snake (*Pantheropsis guttatus*)	A	AMP-**AMC**-AK-S	-	*pic*, *astA*
26	Boa constrictor snake (*Boa constrictor*)	B1	S	-	-
30a	Corn snake (*Pantheropsis guttatus*)	B2	S	-	*irp-2*, *vat*, *traT*, *fyuA*
31a	Corn snake (*Pantheropsis guttatus*)	B1	**S**	*aadA*	*traT*
32a	Milk snake (*Lampropeltis triangulum*)	B2	S	-	*irp-2*, *vat, traT*, *fyuA*
34	Ball python (*Python regius*)	B1	S	-	*traT*
35a	Boa constrictor snake (*Boa constrictor*)	D	S	-	*vat*
36a	Boa constrictor snake (*Boa constrictor*)	A	-	-	*traT*
37a	Corn snake (*Pantheropsis guttatus*)	B1	**CN-AK-S-T-W** ^b^	-	-
39	Corn snake (*Pantheropsis guttatus*)	B1		-	-
41	Milk snake (*Lampropeltis triangulum*)	B2	S	-	*pic*, *irp-2*, *traT*, *fyuA*
47	Corn snake (*Pantheropsis guttatus*)	B1	S	-	-
48	Carpet python (*Morelia spilota*)	B1	**S**	-	*traT*, *fyuA*
54a	Panther chameleon (*Furcifer pardalis*)	B1	S	-	-
46	Central bearded dragon (*Pogona vitticeps*)	A	S-**T**	*tetA*	-
55	Central bearded dragon (*Pogona vitticeps*)	D	**CN-AK-S-T**-CIP	*tetB*, *tetM*	*irp-2*, *fyuA*
61	Central bearded dragon (*Pogona vitticeps*)	B1	**AMP**-S	*blaTEM*	*traT*
62	Central bearded dragon (*Pogona vitticeps*)	B1	S	-	-
63	Central bearded dragon (*Pogona vitticeps*)	B1	S	-	-
44	Green iguana (*Iguana iguana*)	B1	S	-	*traT*
65	Green iguana (*Iguana iguana*)	B1	AMP-S	-	-
17a	Steppe tortoise (*Testudo horsfieldii*)	B1	-	-	*traT*
18a	Steppe tortoise (*Testudo horsfieldii*)	A	**S**	-	*fyuA*
19a	Steppe tortoise (*Testudo horsfieldii*)	A	-	-	-
25a	Red-footed tortoise (*Chelonoidis carbonaria*)	B1	AK-S	-	-
67	Greek tortoise (*Testudo hermanni*)	B1	S	-	-
69	Steppe tortoise (*Testudo horsfieldii*)	B1	S	-	-
70	Steppe tortoise (*Testudo horsfieldii*)	B1	S	-	-
71	Greek tortoise (*Testudo hermanni*)	B1	S	-	*-*

^a^ bold symbols indicate resistance, non-bold symbols indicate intermediate susceptibility; AMP—ampicillin, AMC—amoxicillin/clavulanic acid, AK—amikacin, CIP—ciprofloxacin, CN—gentamicin, S—streptomycin, T—tetracycline, W—trimethoprim; ^b^ MDR strain.

**Table 3:** PCR schemes used to detect virulence genes in *E. coli*.

	Detected Genes	Annealing Temperature (°C)	Reference
Multiplex I	*stx1*, *stx2*, *hylA*, *eaeA*, *saa*	65 (10 cycles) then 62 (20 cycles)	[14]
Multiplex II	*ecsV*, *ent*, *bfpB*, *invE*, *astA*, *aggR*, *pic*, *ipaH*, *elt*, *estIa*, *estIb*	62	[14]
Multiplex III	*ompT*, *iutA*	63	[14]
Multiplex IV	*tsh*, *pap-C*, *iss*, *irp-2*	57	[29]
Multiplex V	*iucD, vat*	54	
Uniplex I	*cva*/*cvi*	58	
Uniplex II	*cnf*	55	[30]
Uniplex III	*kpsII*	56	[4]
Uniplex IV	*traT*	59	
Uniplex V	*fyuA*	59	

## Data Availability

All the data presented in the study are included in the article. Further enquires can be directed to the corresponding author.

## Supplementary Materials

The following are available online at https://www.mdpi.com/article/10.3390/pathogens11020127/s1, Table S1: Sequences of primers used to detect virulence genes, amplicon sizes and references [14,29,30]; Table S2: Sequences of primers used to detect resistance genes [14,23,35,36,37]; Table S3: Primer sequences used to determine phylogenetic groups in *E. coli* strains [35].
