# Supplementary material for: Virulence Profiles and Antibiotic Susceptibility of Escherichia coli Strains from Pet Reptiles
Source: Pathogens. 2022 Jan 21;11(2):127. doi: 10.3390/pathogens11020127 (PMC8880193; doi:10.3390/pathogens11020127)
Supplement: Supplementary file 1 [file pathogens-11-00127-s001.zip › pathogens-1480526-supplementary.pdf]

pathogens

MDPI

Table S1. Sequences of primers used to detect virulence genes, amplicon sizes and references.

Pathovar	Gene	Primers (5′- 3′)	Virulence factor	Amplicon size (bp)	References
EHEC, EPEC	*escV*	ATTCTGGCTCTCTTCTTCTTTATGGCTG<br>CGTCCCCTTTTACAAACTTCATCGC	component EscV	544	[14]
	ent	TGGGCTAAAAGAAGACACACTG<br>CAAGCATCCTGATTATCTCACC	enterotoxin	629	
	eaeA	GACCCGGCACAAGCATAAGC<br>CCACCTGCAGCAACAAGAGG	intimin	384	[14]
EPEC	*bfpB*	GACACCTCATTGCTGAAGTCG<br>CCAGAACACCTCCGTTATGC	bundle-forming pilus	910	[14]
ETEC	*elt*	GAACAGGAGGTTTCTGCGTTAGGTG<br>CTTTCAATGGCTTTTTTTTGGGAGTC	heat labile toxin LT	655	[14]
	estIa	CCTCTTTTAGYCAGACARCTGAATCASTTG<br>CAGGCAGGATTACAACAAAGTTCACAG	heat-stable enterotoxin a	157	
	estIb	TGTCTTTTTCACCTTTCGCTC<br>CGGTACAAGCAGGATTACAACAC	heat-stable enterotoxin b	171	
EIEC	*invE*	CGATAGATGGCGAGAAATTATATCCCG<br>CGATCAAGAATCCCTAACAGAAGAATCAC	invasion protein	766	[14]
	ipaH	GAA AAC CCT CCT GGT CCA TCA GG<br>GCC GGT CAG CCA CCC TCT GAG AGT AC	adhesin siderophore	437	
EHEC	*stx1*	ATA AAT CGC CAT TCG TTG ACT AC<br>AGA ACG CCC ACT GAG ATC ATC	Shiga toxin 1	180	[14]
	stx2	GGCACTGTCTGAAACTGCTCC<br>TCGCCAGTTATCTGACATTCTG	Shiga toxin 2	255	
	hlyA	GCATCATCAAGCGTACGTTCC<br>AATGAGCCAAGCTGGTTAAGCT	hemolysin A	534	
	saa	CGTGATGAACAGGCTATTGC<br>ATGGACATGCCTGTGGCAAC	STEC autoagglutinating adhesin	119	
EAEC	*astA*	TGCCATCAACACAGTATATCCG<br>ACGGCTTTGTAGTCCTTCCAT	EAST1 toxin	102	[14]
	aggR	ACGCAGAGTTGCCTGATAAAG<br>AATACAGAATCGTCAGCATCAGC	transcriptional activator	400	
	pic	AGCCGTTTCCGCAGAAGCC<br>AAATGTCAGTGAACCGACGATTGG	serine protease	1,111	
NTEC	*cnf*	TTATATAGTCGTCAAGATGGA<br>CACTAAGCTTTACAATATTGA	Cytotoxic necrotizing factor	633	[30]
APEC, UPEC	*irp-2*	AAGGATTCGCTGTTACCGGAC<br>AACTCCTGATACAGGTGGC	yersiniabactin synthetase	413	[29]
	iss	ATCACATAGGATTCTGCCG<br>CAGCGGAGTATAGATGCCA	increased serum survival	306	
	pap-C	TGATATCACGCAGTCAGTAGC<br>CCGGCCATATTCACATAA	papACEFG, genes of P fimbriae operon	501	
	tsh	ACTATTCTCTGCAGGAAGTC<br>CTTCCGATGTTCTGAACGT	temperature-sensitive haemagglutinin	824	
	cva/cvi	TGGTAGAATGTGCCAGAGCAAG<br>GAGCTGTTTGTAGCGAAGCC	colicin V	1181	
	iucD	ACAAAAAGTTCTATCGCTTCC<br>CCTGATCCAGATGATGCTC	aerobactin	714	

vat	TCCTGGGACATAATGGTCAG<br>GTGTCAGAACGGAATTGT	vacuolating toxin	981	
ompT	TCATCCCGGAAGCCTCCCTCACTACTAT<br>TAGCGTTTGCTGCACTGGCTTCTGATAC	outer membrane protease T	496	[14]
iutA	GGCTGGACATCATGGGAACTGG<br>CGTCGGGAACGGGTAGAATCG	aerobactin siderophore receptor	302	
fyuA	TGATTAACCCCGCGACGGGAA<br>CGCAGTAGGCACGATGTTGTA	yersiniabactin siderophore receptor	880	[4]
kpsII	GCGCATTTGCTGATACTGTTG<br>CATCCAGACGATAAGCATGAGCA	capsular antigen	272	
traT	GGTGTGGTGCGATGAGCACAG<br>CACGGTTCAGCCATCCCTGAG	complement resistance protein	290	

Table S2. Sequences of primers used to detect resistance genes.

Target gene	Sequence (5′–3′)	Annealing temperature (°C)	Size of PCR product (bp)	Reference
blaTEM	GAG TAT TCA ACA TTT TCG T<br>ACC AAT GCT TAA TCA GTG A	50	445	[35]
blaCTX	CGCCGCATACACTATTCTCAGAATGA<br>ACGCTCACCGGCTCCAGATTTAT		593	
blaOXA	ATGTGCAGyACCAGTAArGTkATGGC<br>TGGGTrAArTArGTsACCAGAAyCAGCGG		813	
blaSHV	ACACAATACATATCAACTTCGC<br>AGTGTGTTTAGAATGGTGATC		237	
tetA	GTG AAA CCC AAC ATA CCC C<br>GAA GGC AAG CAG GAT GTA G	63	887	[36]
tetB	CCT TAT CAT GCC AGT CTT GC<br>ACT GCC GTT TTT TCG CC		773	
tetC	GCTGTAGGCATAGGCTTGGT<br>GCCGGAAGCGAGAAGAATCA		888	
tetM	GTG GAC AAA GGT ACA ACG AG<br>CGG TAA AGT TCG TCA CAC AC	62	406	[23]
tetO	AAC TTA GGC ATT CTG GCT CAC<br>TCC CAC TGT TCC ATA TCG TCA		515	
tetK	GAT CAA TTG TAG CTT TAG GTG AAG G<br>TTT TGT TGA TTT ACC AGG TAC CAT T		155	
tetL	TGG TGG AAT GAT AGC CCA TT<br>CAG GAA TGA CAG CAC GCT AA		229	
aadA	GTGGATGGCGGCCTGAAGCC<br>AATGCCCAGTCGGCAGCG	63	525	[36]
strA/strB	ATGGTGGACCCTAAAACTCT<br>CGTCTAGGATCGAGACAAAG		893	
aac(3)-II	ATA TCG CGA TGC ATA CGC GG<br>GAC GGC CTC TAA CCG GAA GG	56	877	[14]
aac(3)-IV	CTT CAG GAT GGC AAG TTG GT<br>TCA TCT CGT TCT CCG CTC AT	55	286	[14]
aphA1	ATG GGC TCG CGA TAA TGT C<br>CTC ACC GAG GCA GTT CCA T	50	600	
aphA2	GAA CAA GAT GGA TTG CAC GC<br>GCT CTT CAG CAA TAT CAC GG	50	680	
sul1	CGGCGTGGGCTACCTGAACG	65	433	[36]

	GCCGATCGCGTGAAGTTCCG			
sul2	CGG CAT CGT CAA CAT AAC CT<br>TGTGCGGATGAAGTCAGCTC		721	
sul3	CAA CGG AAG TGG GCG TTG TGG A<br>GCT GCA CCA ATT CGC TGA ACG		244	
dfrA1	TGGTAGCTATATCGAAGAATGGAGT<br>TATGTTAGAGGCGAAGTCTTGGGTA		425	
dfrA5	AGCTACTCTTTAAAGCCTTGACGTA<br>GTGTTGCTCAAAAACAACTTCG	60	341	[37]
dfrA7-17	ACATTTGACTCTATGGGTGTTCTTC<br>AAAACTGTTCAAAAACCAAATTGAA		280	

Table S3. Primer sequences used to determine phylogenetic groups in *E. coli* strains.

Gene	Sequence of primers (5′-3′)	PCR product	Amplicon size [bp]	Reference
yjaA	TGAAGTGTCAGGAGACGCTG<br>TGAAGTGTCAGGAGACGCTG	stress response protein	211	
TspE4.C2	GAG TAA TGT CGG GGC ATT CA<br>CGC GCC AAC AAA GTA TTA CG	anonymous DNA fragment	152	
chuA	GAC GAA CCA ACG GTC AGG AT<br>TGC CGC CAG TAC CAA AGA CA	outer membrane hemin receptor ChuA	279	[35]
svg	TCCGGCTGATTACAAACCAAC<br>CTGCACGAGGTTGTAGTCCTG	hypothetical protein, sequence located at open reading frame (ORF) specific for virulent $B2_1$ subgroup of *E. coli*	434	
uidA	ATC ACC GTG GTG ACG CATGTC GC<br>CAC CAC GAT GCC ATG TTC ATC TGC-3	glucuronidase	486	
